# Supplementary material for: Probiotic Supplements Effect on Feeding Tolerance, Growth and Neonatal Morbidity in Extremely Preterm Infants: A Systematic Review and Meta-Analysis
Source: Nutrients. 2025 Apr 1;17(7):1228. doi: 10.3390/nu17071228 (PMC11990243; doi:10.3390/nu17071228)
Supplement: Supplementary file 1 [file nutrients-17-01228-s001.zip › nutrients-3552965-supplementary.pdf]

Supplemental Information

Supplemental file 1: List of all full-text screened studies including reason of exclusion

Table S1. Inclusion and exclusion criteria 1

Table S2. Search terms 3

Table S3. Risk of bias for included studies........ 7

Table S1. Inclusion and exclusion criteria

	Criteria	
**Category**	**Inclusion**	**Exclusion**
Population	Extremely preterm infants (born in gestational week 22+0 – 27+6)<br>Very low birth weight (<1500 grams)<br>Extremely low birth weight (<1000 grams)	Moderate or late preterm infants (born in gestational week (32-37)<br>Birth weight >1500 grams
Interventions	Probiotics<br>Multi strain and single strain	Prebiotics
Control intervention	Placebo<br>No treatment/usual care if no placebo-controlled study is available	Different treatments
Outcomes	Feeding intolerance<br>Time to full enteral feeds	Studies that do not include at least one of the outcomes listed under the inclusion criteria

	Growth (head circumference, length, weight, standard deviation, Z score, small for gestational age)<br>Necrotizing enterocolitis<br>Sepsis (late onset sepsis and culture-proves sepsis)<br>All-cause mortality<br>Length of hospitalization<br>Adverse events (general)	
Study design	RCTs<br>Nonrandomized controlled trials (including observational controlled studies with a concurrent control group)	Case-control studies<br>Case series<br>Case reports<br>Pre-post implementation studies<br>Nonsystematic reviews<br>Studies without a control group<br>Systematic reviews and meta-analyses
Geography	No limitations	
Language	No limitations	

Table S2. Search terms

Search	**Medline -** June 13, 2023	Results
#1	Probiotics/	23908
#2	exp Lactobacillus/	24635
#3	Limosilactobacillus reuteri/	1020
#4	exp Bifidobacterium/	7259
#5	Saccharomyces boulardii/	197
#6	Streptococcus thermophilus/	1009
#7	exp Enterococcus/	21887
#8	(bifidobacterium or enterococcus or lactobacillus or limosilactobacillus reuteri or L reuteri or probiotic* or pro-biotic* or saccharomyces boulardi* or s boulardi* or streptococcus thermophilus).ti,ab,kf.	97577
#9	or/1-8	113581
#10	exp Infant, Premature/	64737
#11	Premature Birth/	20999
#12	exp Infant, Very Low Birth Weight/	11838
#13	(low birth weight or low birthweight or prematurity).ti,ab,kf.	62588
#14	((premature* or preterm* or pre-term*) and (birth* or infant* or infancy or neonat* or newborn* or new-born*)).ti,ab,kf.	123508
#15	or/10-14	174006
#16	9 and 15	1598
#17	exp Clinical Trial/	972268
#18	Epidemiologic Studies/	9344
#19	exp Case-Control Studies/	1422232
#20	Controlled Before-After Studies/	726
#21	exp Cohort Studies/	2490369
#22	Cross-Sectional Studies/	469048
#23	Observational Study/	142768
#24	drug therapy.fs.	2598995
#25	(case-control or cohort* or control group or control* before-after or cross section* or epidemiolog* or follow up or followup or groups or longitudinal* or observation* or placebo or prospectiv* or random* or trial or retrospectiv*).ti,ab.	7678801

#26	(phase I or phase II or phase III or phase IV or phase 1 or phase 2 or phase 3 or phase 4 or phase one or phase two or phase three or phase four).ti,ab.	180765
#27	or/17-26	10206034
#28	exp animals/ not humans.sh.	5130428
#29	27 not 28	9181521
#30	16 and 29	944
**Search**	**Embace** - June 13, 2023	**Results**
#27	#26 AND ('article'/it OR 'article in press'/it OR 'conference paper'/it OR 'erratum'/it OR 'preprint'/it)	1053
#26	#25 NOT ([animals]/lim NOT [humans]/lim)	1727
#25	#15 AND #24	1798
#24	#16 OR #17 OR #18 OR #19 OR #20 OR #21 OR #22 OR #23	11613999
#23	'phase i':ti,ab OR 'phase ii':ti,ab OR 'phase iii':ti,ab OR 'phase iv':ti,ab OR 'phase 1':ti,ab OR 'phase 2':ti,ab OR 'phase 3':ti,ab OR 'phase 4':ti,ab OR 'phase one':ti,ab OR 'phase two':ti,ab OR 'phase three':ti,ab OR 'phase four':ti,ab	346130
#22	'case control':ti,ab OR cohort*:ti,ab OR 'control group':ti,ab OR 'control* before-after':ti,ab OR 'cross section*':ti,ab OR epidemiolog*:ti,ab OR 'follow up':ti,ab OR followup:ti,ab OR groups:ti,ab OR longitudinal*:ti,ab OR observation*:ti,ab OR placebo:ti,ab OR prospectiv*:ti,ab OR random*:ti,ab OR trial:ti,ab OR retrospectiv*:ti,ab	10737068
#21	'observational study'/de	323178
#20	'cross-sectional study'/de	559688
#19	'cohort analysis'/de	1005654
#18	'case control study'/exp	220452
#17	'epidemiology'/de	256785
#16	'clinical trial'/exp	1823492
#15	#9 AND #14	3174
#14	#10 OR #11 OR #12 OR #13	245056
#13	(premature*:ti,ab,kw OR preterm*:ti,ab,kw OR 'pre term*':ti,ab,kw) AND (birth*:ti,ab,kw OR infant*:ti,ab,kw OR infancy:ti,ab,kw OR neonat*:ti,ab,kw OR newborn*:ti,ab,kw OR 'new born*':ti,ab,kw)	169381
#12	'low birth weight':ti,ab,kw OR 'low birthweight':ti,ab,kw OR prematurity:ti,ab,kw	85568
#11	'very low birth weight'/exp	17577
#10	'prematurity'/exp	133981
#9	#1 OR #2 OR #3 OR #4 OR #5 OR #6 OR #7 OR #8	177480

#8	bifidobacterium:ti,ab,kw OR enterococcus:ti,ab,kw OR lactobacillus:ti,ab,kw OR 'limosilactobacillus reuteri':ti,ab,kw OR 'l reuteri':ti,ab,kw OR probiotic*:ti,ab,kw OR 'pro biotic*':ti,ab,kw OR 'saccharomyces boulardi*':ti,ab,kw OR 's boulardi*':ti,ab,kw OR 'streptococcus thermophilus':ti,ab,kw	117646
#7	'enterococcus'/exp	65568
#6	'streptococcus thermophilus'/de	3581
#5	'saccharomyces boulardii'/de	1973
#4	'bifidobacterium'/exp	21051
#3	'lactobacillus reuteri'/de	3588
#2	'lactobacillus'/exp	52994
#1	'probiotic agent'/exp	51634
**Search**	**Cochrane Library -** June 13, 2023	**Results**
#1	[mh ^Probiotics]	2870
#2	[mh Lactobacillus]	1547
#3	[mh ^"Limosilactobacillus reuteri"]	187
#4	[mh Bifidobacterium]	1118
#5	[mh ^"Saccharomyces boulardii"]	57
#6	[mh ^"Streptococcus thermophilus"]	87
#7	[mh Enterococcus]	427
#8	(bifidobacterium:ti,ab,kw OR enterococcus:ti,ab,kw OR lactobacillus:ti,ab,kw OR "limosilactobacillus reuteri":ti,ab,kw OR "L reuteri":ti,ab,kw OR probiotic*:ti,ab,kw OR pro-biotic*:ti,ab,kw OR ("saccharomyces" NEXT boulardi*):ti,ab,kw OR ("s" NEXT boulardi*):ti,ab,kw OR "streptococcus thermophilus":ti,ab,kw)	13161
#9	#1 OR #2 OR #3 OR #4 OR #5 OR #6 OR #7 OR #8	13166
#10	[mh "Infant, Premature"]	4885
#11	[mh ^"Premature Birth"]	2082
#12	[mh "Infant, Very Low Birth Weight"]	1181
#13	("low birth weight":ti,ab,kw OR "low birthweight":ti,ab,kw OR prematurity:ti,ab,kw)	11844
#14	((premature*:ti,ab,kw OR preterm*:ti,ab,kw OR pre-term*:ti,ab,kw) AND (birth*:ti,ab,kw OR infant*:ti,ab,kw OR infancy:ti,ab,kw OR neonat*:ti,ab,kw OR newborn*:ti,ab,kw OR new-born*:ti,ab,kw))	20700
#15	#10 OR #11 OR #12 OR #13 OR #14	24155

#16	#9 AND #15	555
#17	#9 AND #15 in Trials	541
**Search**	**Web of Science Core Collection - June 13, 2023**	**Results**
#1	TS=(bifidobacterium OR enterococcus OR lactobacillus OR "limosilactobacillus reuteri" OR "L reuteri" OR probiotic* OR pro-biotic* OR "saccharomyces boulardi*" OR "s boulardi*" OR "streptococcus thermophilus")	134808
#2	TS=("low birth weight" OR "low birthweight" OR prematurity)	70518
#3	TS=((premature* OR preterm* OR pre-term*) AND (birth* OR infant* OR infancy OR neonat* OR newborn* OR new-born*))	135443
#4	#2 OR #3	170363
#5	#1 AND #4	1809
#6	TI=(case-control OR cohort* OR "control group" OR "control* before-after" OR "cross section*" OR epidemiolog* OR "follow up" OR followup OR groups OR longitudinal* OR observation* OR placebo OR prospectiv* OR random* OR trial OR retrospectiv*) OR AB=(case-control OR cohort* OR "control group" OR "control* before-after" OR "cross section*" OR epidemiolog* OR "follow up" OR followup OR groups OR longitudinal* OR observation* OR placebo OR prospectiv* OR random* OR trial OR retrospectiv*)	9768924
#7	TI=("phase I" OR "phase II" OR "phase III" OR "phase IV" OR "phase 1" OR "phase 2" OR "phase 3" OR "phase 4" OR "phase one" OR "phase two" OR "phase three" OR "phase four") OR AB=("phase I" OR "phase II" OR "phase III" OR "phase IV" OR "phase 1" OR "phase 2" OR "phase 3" OR "phase 4" OR "phase one" OR "phase two" OR "phase three" OR "phase four")	257433
#8	#7 OR #6	9880705
#9	#5 AND #8	925
**Search**	**Web of Science Core Collection - June 13, 2023**	**Results**
	**Condition or disease**: prematur* or preterm* or pre-term* or "low birth weight" OR "low birthweight"<br><br>**Other terms**: bifidobacterium OR enterococcus OR lactobacillus OR "limosilactobacillus reuteri" OR "L reuteri" OR probiotic* OR pro-biotic* OR "saccharomyces boulardi*" OR "s boulardi*" OR "streptococcus thermophilus"	19

Table S3. Risk of bias for included studies

Risk of bias non-randomized studies							
**Author, year**	**Bias due to confound-ding**	**Bias in selection of participants into the study**	**Bias in classification of interventions**	**Bias due to deviations from intended interventions**	**Bias due to missing data**	**Bias in measurement of outcomes**	**Bias in selection of the reported result**	**Overall risk of bias**
Chang 2022	Moderate	Low	Low	Low	Low	Moderate	Low	Serious
Härtel 2017	Low	Low	Low	Low	Low	Moderate	Low	Moderate

Risk of bias, randomized controlled trials						
**Author, year**	**Domain 1. Randomization process**	**Domain 2. Deviations from intended interventions**	**Domain 3. Missing outcome data**	**Domain 4. Measurement of the outcome**	**Domain 5. Selection of the reported result**	**Overall risk of bias**
Al-Hosni 2012	Low	Low	Low	Low	Low	Low
Alshaikh 2022	Low	Moderate	Low	Moderate	Low	Moderate
Costeloe 2016	Low	High	Low	Low	Low	High
Dobryk 2023	Low	Moderate	Low	Moderate	Low	Moderate
Guney-Varal 2017	Moderate	Moderate	Low	Moderate	Moderate	Moderate
Havranek 2013	Low	Low	Moderate	Low	Moderate	Moderate
Jacobs 2013	Low	Low	Low	Low	Low	Low
Manzoni 2009	Low	Low	Low	Low	Low	Low
Patole 2014	Low	Low	Low	Low	Low	Low
Totsu 2014	Moderate	Low	Moderate	Low	Low	Moderate
Wejryd 2019	Low	Low	Low	Low	Low	Low
